# In vitro degradation, haemolysis and cytotoxicity study of Mg‐0.4Ce/ZnO_2_ nanocomposites

**DOI:** 10.1049/nbt2.12032

**Published:** 2021-03-22

**Authors:** Meenachi Prabakaran, Subashini Rajakannu, Lakshminarayanan K Adhimoolam, Manoj Gupta

**Affiliations:** ^1^ Department of Biomedical Engineering SSN College of Engineering Chennai India; ^2^ Department of Mechanical Engineering SSN College of Engineering Chennai India; ^3^ Department of Mechanical Engineering National University of Singapore Singapore Singapore

## Abstract

Magnesium is an ideal candidate for biodegradable implants, but the major concern is its uncontrollable degradation for application as a biomaterial. The in vitro corrosion and cytotoxicity of Mg‐0.4Ce/ZnO_2_ (magnesium nanocomposites) were studied to determine its suitability as a biodegradable material. The polycrystalline nature of Mg‐0.4Ce/ZnO_2_ was assessed using an optical microscope. The hydrophobic nature of Mg‐0.4Ce/ZnO_2_ was determined by contact angle measurements. The corrosion resistance of magnesium nanocomposites was tested in phosphate buffer solution (PBS) and it was improved by the gradual deposition of a protective layer on its surface after 48 h. The cytotoxicity of Mg‐0.4Ce/ZnO_2_ was evaluated by 3‐(4,5‐dimethylthiazol‐2‐yl)‐2,5‐diphenyltetrazolium bromide (MTT) assay and calcium deposition by Alizarin red staining using sarcoma osteogenic (Saos2) cells. The haemocompatibility test of Mg‐0.4Ce/ZnO_2_ showed 30% haemolysis, which is higher than the safe value for biomaterials, and cell viability was reduced after 24 h in comparison with control groups. The calcium deposition by sarcoma osteogenic cells showed a brick red colour deposition in both the control group and Mg‐0.4Ce/ZnO_2_ after 24 h. The preliminary degradation results of Mg‐0.4Ce/ZnO_2_ showed good corrosion resistance; however further improvement is needed in haemolysis and cytotoxicity studies for its use as a biodegradable material for orthopaedic applications.

## 1 INTRODUCTION

Ageing of the global population has increased public concern regarding health and made huge demands for degradable implants [1]. Existing metallic implants made of stainless steel, titanium alloys (Ti alloys) and cobalt–chromium alloys (Co‐Cr alloys) are widely used in orthopaedic applications to assist the repair or replacement of bone tissue. These implants require second surgery once the tissue has healed, which leads to an increase in the healthcare costs and patient morbidity [2]. Magnesium has become the primary focus of investigation as a biodegradable implant. It has mechanical properties (Young's modulus and density) comparable to those of human bone and has shown stimulatory effects on the growth of new bone tissue, which make it an attractive material for orthopaedic applications [3, 4, 5, 6]. In addition, it is also an important component of 300 enzymes that act as catalysts [7]. The practical limitations of magnesium include its uncontrolled degradation because of the lowest standard potential of −2.38 V of all engineering metals. Therefore, the inadequacy of being a short‐term support can lead to premature loss of mechanical strength and structural integrity, which can eventually result in implant failures [8]. This uncontrolled degradation has hindered the widespread use of Mg‐based materials for implant applications. It has been reported that the study of Mg based alloys with different metals of varied concentrations shown to be less toxic to the environment of the host tissue and also enhances the mechanical properties [9, 10, 11]. The Mg alloys for orthopaedic applications with optimisation of implant osseointegration (implant ability to integrate with bone tissue) are still under investigation [12, 13, 14]. The uncontrolled degradation problems have been successfully addressed through the addition of various alloying elements and rare earth (RE) elements, and have increased their use, as these impart significant property improvements [15]. Such persistent metallurgical investigations routinely produce Mg alloys with improved properties that are suitable for biomedical applications. The continued drive to develop lightweight materials suitable for biomedical use has resulted in a renewed focus on magnesium due to its matching density with bone and biodegradability. Within the last decade, attention has focused on the use of nanoparticle reinforcements in both pure Mg and Mg alloys [16]. Previous reports stated that the nanocomposite materials act as an alternative to overcome the limitations of microcomposites and monolithic materials [17]. Nanocomposites have been reported to be the materials of the 21st century with unique design and property combinations that are not found in conventional composites and existing implants [18]. Over the last two decades several processing techniques have been developed to fabricate nanocomposites and these are being widely used in many applications around the world [19].

In the present study, the new Mg 0.4%Ce/ZnO_2_ nanocomposites were produced by the cost‐effective disintegrated melt deposition technique [20]. The effect of rare earth elements such as cerium (Ce) on fibroblast and osteoblast proliferation and differentiation has been studied and it has been demonstrated that an appropriate percentage of it can improve the mechanical properties, corrosion behaviour, and biocompatibility. Ce has the ability to replace the calcium ions in biomolecules without necessarily substituting for their functionality [21]. However, the biocompatibility of rare earth element is a major concern. Nanoparticles of noble metals, with the advancement of new materials, have been successfully developed for different engineering and biological science purposes. ZnO NPs possess antimicrobial (both antibacterial and antifungal) properties that have attracted significant attention, especially in the field of biomedical application [22].

In the present investigation, the feasibility of magnesium nanocomposites (Mg‐0.4Ce –ZnO_2_) as a biodegradable implant is investigated through microstructural observation, biodegradation study of Mg‐0.4Ce/ZnO_2_, and haemocompatibility and cytotoxicity assessment.

## 2 MATERIALS AND METHODS

### 2.1 Preparation of specimens

Partially or fully characterised nanocomposite Mg‐0.4Ce/ZnO_2_ were received from Dr. M. Gupta, Associate Professor, Department of Mechanical Engineering, National University of Singapore. These nanocomposites were prepared through the disintegrated melt deposition technique using high‐purity Mg, Ce, and ZnO_2_ [20]. Then, this material was cut into discs of 4 mm using an electrical discharge machine (Ratnaparkhi SC100). For biocompatibility evaluation, all specimens were sterilised under UV lamps for 2 h.

### 2.2 Microstructure characterisation

The microstructure of Mg‐0.4Ce/ZnO_2_ was examined using an optical microscope (Olympus, Japan, BX51M‐N33MD). The specimens were mounted, mechanically ground, and polished with silicon carbide paper up to 1500 grit size. The specimens were then etched with a solution containing 10 ml acetic acid, 4.2 g picric acid, 10 ml deionised water and 70 ml ethanol for 5–10 s [23]. An Olympus metallurgical microscope was used for microstructure observation.

### 2.3 Measurement of contact angle

To determine the wettability of the sample, the contact angles of Mg‐0.4Ce/ZnO_2_ were obtained by the sessile drop method using water. The tests were conducted under ambient conditions at various locations on the specimen with sufficient spacing to prevent any interference [24].

### 2.4 Haemocompatibility assay

In order to evaluate the haemocompatibility of Mg‐0.4Ce/ZnO_2_, fresh human blood and sodium citrate (3.8 wt.%) in the ratio of 9:1 was taken and diluted with normal saline (4:5 ratio by volume) [25]. Samples were soaked in 10 ml normal saline tubes and kept at 37°C for 30 min. Then, 0.2 ml of diluted blood was added to each tube and the mixture was incubated for 60 min at 37°C. Ten ml normal saline solution and 10 ml deionised water were used as negative and positive controls, respectively. Then, the tubes were centrifuged at 3000 rpm for 5 min. The optical density (OD) of the clear supernatant was detected using a spectrophotometer at the wavelength of 545 nm. The haemolysis ratio is expressed as a percentage and is calculated using the formula:

$$HR=\left[OD_{t}-OD_{n}\right]/\left[OD_{p}-OD_{n}\right]*100$$

where *HR* is the haemolysis ratio (%)

*OD*_*t*_ is the OD value of the tested group (%)

*OD*_*n*_ is the OD value of the negative control (%)

*OD*_*p*_ is the OD value of the positive control (%).

### 2.5 Biodegradation study of Mg‐0.4Ce/ZnO_2_

The in vitro degradation test was carried out at 37^o^C for 14 days. Phosphate buffer solution (PBS) is a saline solution containing 8 g sodium chloride (NaCl), 0.2 g potassium chloride (KCl), 1.5 g disodium phosphate (Na_2_HPO_4_), and 0.2 g potassium dihydrogen phosphate (KH_2_PO_4_) and is adjusted to a pH of 7.4 in order to closely simulate the physiological environment (all the chemicals were purchased from Sigma Aldrich). PBS was chosen in order to determine the effects of Mg‐0.4Ce/ZnO_2_ in an aggressive physiological environment. The study was carried out under static conditions (at 37^o^C) and the samples were immersed in PBS solution for 14 days. After 1, 3, 5, 7, and 14 days the samples were removed from the solution, gently rinsed with distilled water, and allowed to air dry until the sample reached a constant mass [26]. Degradation products that precipitated on the surface of the samples were left intact, while soluble degradation products were allowed to remain at the bottom of the solution. The degradation rate (mm/year) was calculated using the following formula:

$$\mathrm{Degradation}\;\mathrm{rate}=\frac{\mathrm{Initial}\;\mathrm{weight}-\mathrm{Final}\;\mathrm{weight}}{\mathrm{Initial}\;\mathrm{weight}}*100$$

### 2.6 Cytotoxicity assessment

#### 2.6.1 Cell culture and seeding

The cancer cell lines were obtained from the National Centre for Cell Science (NCCS), Pune, India. The cells were maintained in Minimal Essential Media supplemented with 10% FBS, penicillin (100 U/ml), and streptomycin (100 µg/ml) in 5% CO_2_ at 37°C. Cells were seeded at 5000 cells/well in 96‐well plates and the samples were incubated.

#### 2.6.2 MTT assay

Sarcoma osteogenic (Saos2) cells were chosen to assess the cytotoxicity of Mg‐0.4Ce/ZnO_2_ [27, 28, 29]. The samples were then transferred to the 96‐well plates using plastic forceps without touching the top surface of the material. The cells without the sample served as controls. After incubating the cells in a humidified atmosphere of 5% CO_2_ at 37^o^C for 1 day, 50 µl of MTT (3‐[4,5‐dimethylthiazol‐2‐yl]‐2,5‐diphenyltetrazolium bromide) solution (5 mg/ml in test medium) was added to each well and incubated at 37^o^C for 4 h [24, 25, 26]. Then, the medium was aspirated and replaced by 100 µl dimethyl sulphoxide in each well and mixed thoroughly to dissolve the dark blue crystals. After 10 min at room temperature, when all the crystals were ensured to be dissolved, the samples were removed from the wells and the 24‐well plates were read on a Microplate reader (Model 550, Bio Rad Corp) at a test wavelength of 570 nm. For the MTT assay, triplicates were performed and the cell viability was calculated according to the following formula:

Cellviability(%)=ODtest/ODcontrol∗100

where OD means optical density.

#### 2.6.3 Biomineralisation by Alizarin red staining (ARS)

Deposition of calcium on Saos2 cells was determined by Alizarin red staining. The ARS staining dye binds with calcium produced by the Saos2 cells [30]. The samples with cells were washed using PBS three times and fixed with 4% formaldehyde for 15 min at room temperature. Then, the cells were washed three times carefully with distilled water and stained with ARS (40 mM) for 30 min at room temperature. After washing three times with distilled water to remove excess dye, the cells were observed under a fluorescence microscope (Magnus, MLXI‐TR).

## 3 RESULTS AND DISCUSSION

### 3.1 Microstructure characterisation

**FIGURE 1:**
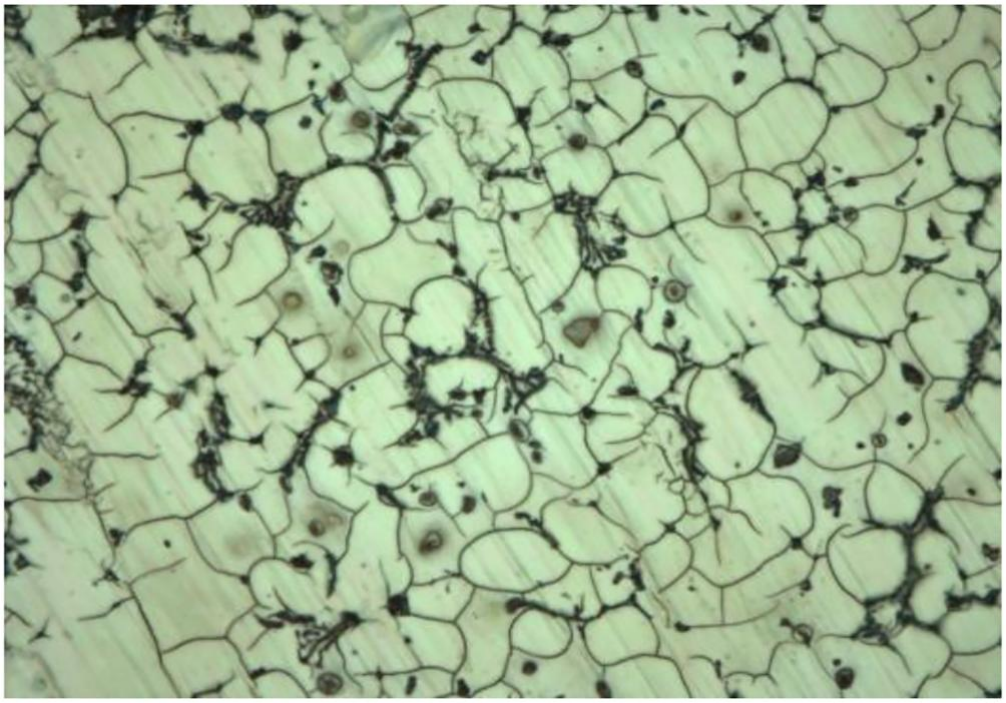
Micrograph of Mg‐0.4Ce/ZnO_2_ (50×)

The microstructure of Mg‐0.4Ce/ZnO_2_ as polycrystalline in nature is shown in Figure 1 [20]. The nanocomposites were found to be distributed along the grain boundaries and were found to be stable. Nanocomposite addition led to the formation of thin lamellar eutectic at the grain boundaries. The addition of zinc oxide nanoparticles and cerium was found to refine the microstructure.

### 3.2 Measurement of contact angle

**FIGURE 2:**
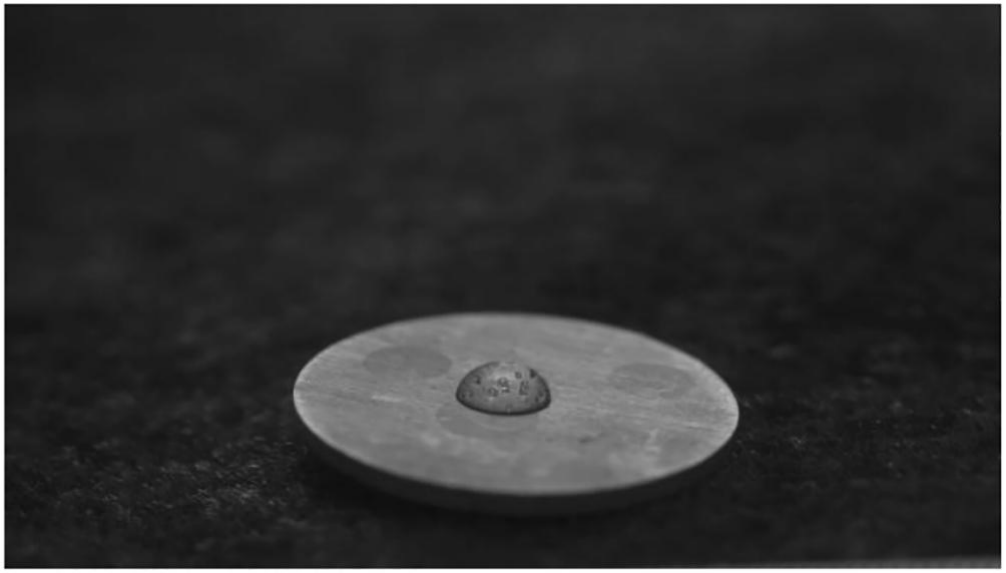
Contact angle of Mg‐0.4Ce/ZnO_2_

In the current investigation, the contact angle of Mg‐0.4Ce/ZnO_2_ was measured to be 71° as shown in Figure 2 and the surface was found to be hydrophilic. The standard range of contact angle for a hydrophobic and hydrophilic surface was greater than 90° and less than 90°, respectively. Arima and Iwata (2007) reported that HUVECs and HeLa cells adhered well on moderately wettable surfaces with water contact angles of 40°–60° [31].

### 3.3 Haemocompatibility assay

The rate of haemolysis has been assessed for the determination of haemocompatibility of biomaterials. According to ISO 10993‐4:2017 [32], the haemolysis rate of the biomaterials should be less than 5%. In the present study, the haemolysis rate of Mg‐0.4Ce/ZnO_2_ was found to be 30%, which is indicative of severe haemolysis. Haemolysis is the process of haemoglobin release from erythrolysis. The haemolysis test is based on the degree to which the erythrolysis and haemoglobin dissociate when the material comes into contact with erythrocytes in vitro. The addition of rare earth elements (RE) in Mg may have caused small deleterious effects on haemolysis. Additional factors influencing haemolysis include hydrogen evolution, change in pH value, and the concentration of released ions [24]. From the results of the current study it was found that Mg‐0.4Ce/ZnO_2_ does not meet the requirement for biomaterials, and that an additional requirement is needed to prevent haemolysis.

### 3.4 Biodegradation study of Mg‐0.4Ce/ZnO_2_

**FIGURE 3:**
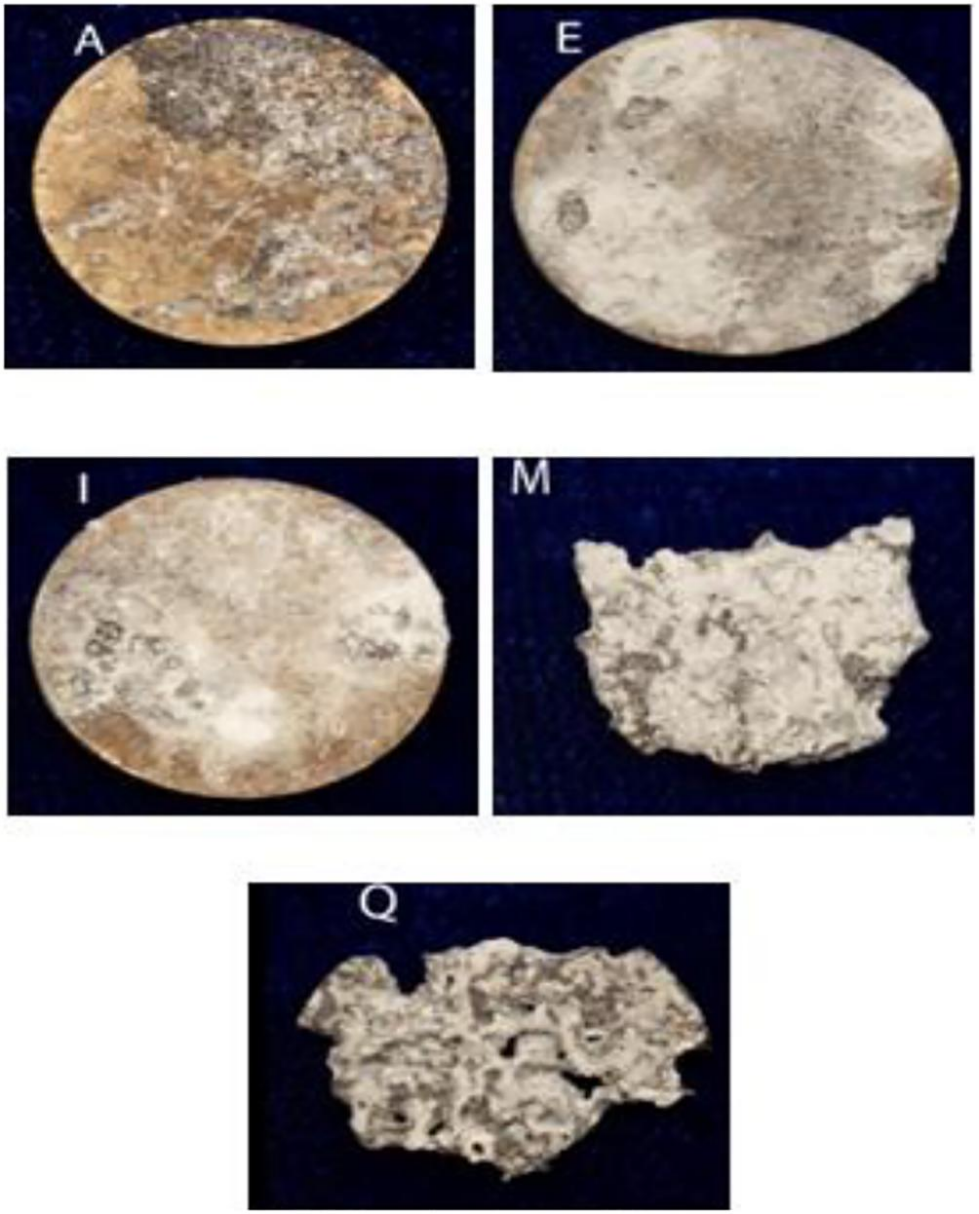
The rate of degradation of Mg‐0.4Ce/ZnO_2_ after the first (A), third (E), fifth (I), seventh (M), and 14th days (Q)

Figure 3 demonstrates the surface morphology of Mg‐0.4Ce/ZnO_2_ samples after immersion in PBS at 37°C for 1, 3, 5, 7, and 14 days. At the initial stage of immersion (Figure 3a) the protection layer was formed on the surface of Mg‐0.4Ce/ZnO_2_ [33]. When the immersion time was increased, the protective layer formed on Mg‐0.4Ce/ZnO_2_ could protect the substrate only for a certain period because of its very thin layer. During the initial period of immersion, the surface was protected, and localised degradation was restrained.

**FIGURE 4:**
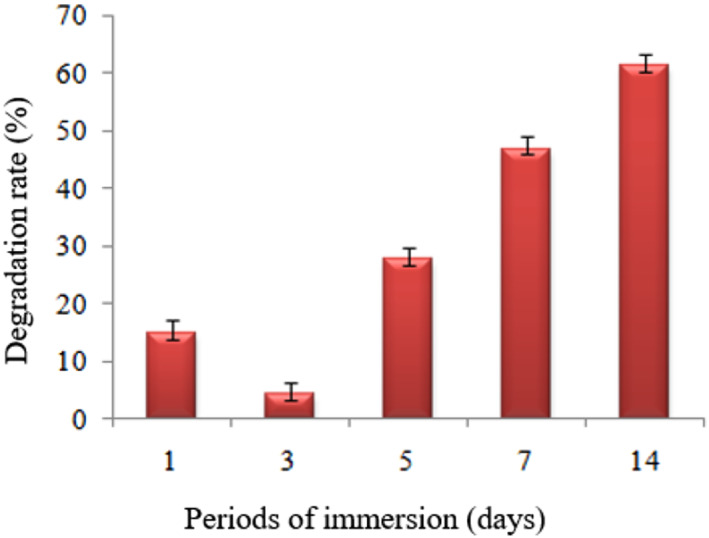
The rate of degradation of Mg‐0.4Ce/ZnO_2_ after the first, third, fifth, seventh and 14th days of exposure in phosphate buffer solution

It was found that when the chloride concentration in the corrosion environment rose, magnesium hydroxide started to be converted into highly soluble magnesium chloride and severe corrosion occurred on the substrate [34]. On the third day, a dense white product appeared on one side of the sample, along with the development of shallow pits, as shown in Figure 3e. The depth of pits progressed across the side surface of the sample by the fifth day, as shown in Figure 3i. The protective layer appeared to be white and relatively inhomogeneous at visual inspection, from the first to fifth days. Although the protective layer covered the entire sample surface on the fifth day, the variation in sample size was negligible. The loss of a protective layer on certain areas of the surface was encouraged, by the localised corrosion, to propagate [33]. Because of the stable protective layer, ions in solution took a longer time to penetrate the surface. The delamination of the sample surface started from the edges and then slowly migrated inward while leaving behind a rough contour on the seventh day as shown in Figure 3M. The degradation layer was rough, non‐porous and heterogeneous, and it migrated inward from the edge until it covered the entire surface. Since the degradation was not uniform, the sample surface started to shed fragments and lost its structural integrity at the end of the seventh day. Most of the visible degradation on the sample surface occurred between 7 and 14 days. Eventually, the sample surface was found to be porous and broke into fragments because of the propagation of localised corrosion, and this became too severe to keep the protective layer intact by the 14th day, as shown in Figure 3Q. Figure 4 illustrates the degradation rate in terms of weight loss of the samples in PBS. The mass of the samples was constant on the first day of immersion, followed by a slight mass gain on the third day due to the white deposit, a slight mass loss on the fifth day, and then slow yet continuing mass loss between the seventh and 14th days [35, 36]. The sample lasted longer in PBS, possibly because of the more stable protective layer formed on the surface by incorporating ions from the PBS. The samples were tested under static conditions for a short period of time. This process would be totally different in vivo as the dynamic circulation system will remove the degradation products and prevent the increase in local pH. Therefore, the degradation products can be eliminated quickly from the body and therefore only low levels of ions will be absorbed.

### 3.5 Cytotoxicity assessment

**FIGURE 5:**
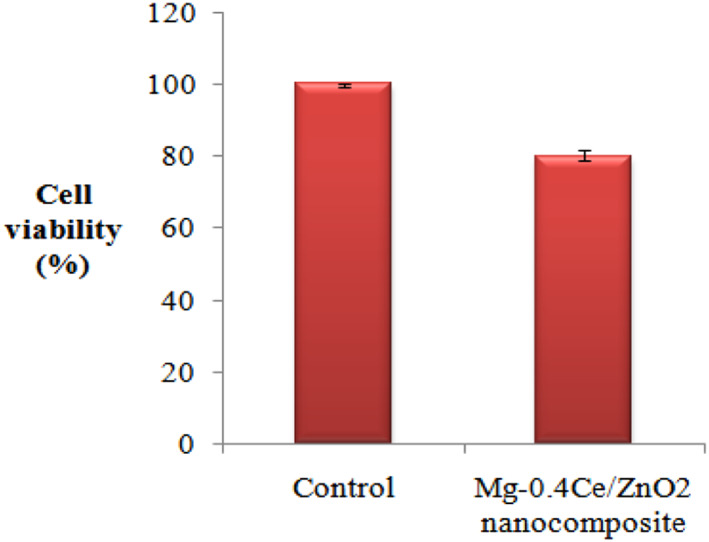
Viability of Saos2 cells in direct contact with Mg‐0.4Ce/ZnO_2_ after 24 h

**FIGURE 6:**
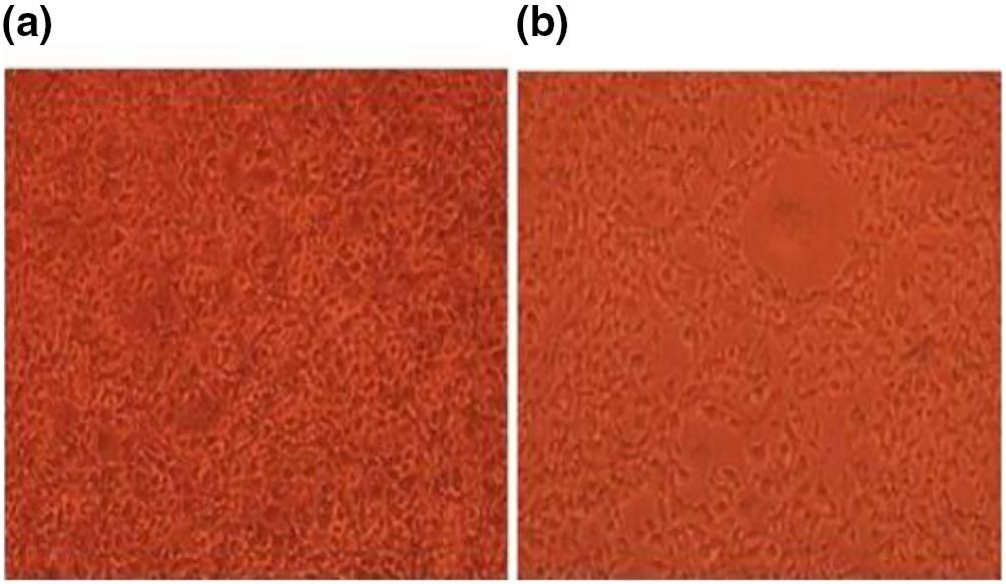
Saos2 cells metabolism in direct contact with Mg‐0.4Ce/ZnO_2_ after 24 h: (a) control; (b) Mg‐0.4Ce/ZnO_2_

Figure 5 depicts the viability of Saos2 cells for control and Mg‐0.4Ce/ZnO_2_ after 1 day of culture. The control group showed higher cell viability than Mg‐0.4Ce/ZnO_2_, as shown in Figure 6a. The reduction in viability of Saos2 cells was due to an increase in pH, as shown in Figure 6b. The pH of Mg‐0.4Ce/ZnO_2_ was measured to be 8.4. Direct MTT test revealed that there is a reduction in the relative growth rate by 20% after 1 day for Mg‐0.4Ce/ZnO_2_ under static culture [37]. The results of MTT assay for the Mg‐0.4Ce/ZnO_2_ surface appear to have a cytotoxicity effect after 1 day due to an increase in pH.

### 3.6 Biomineralisation by Alizarin red staining (ARS)

**FIGURE 7:**
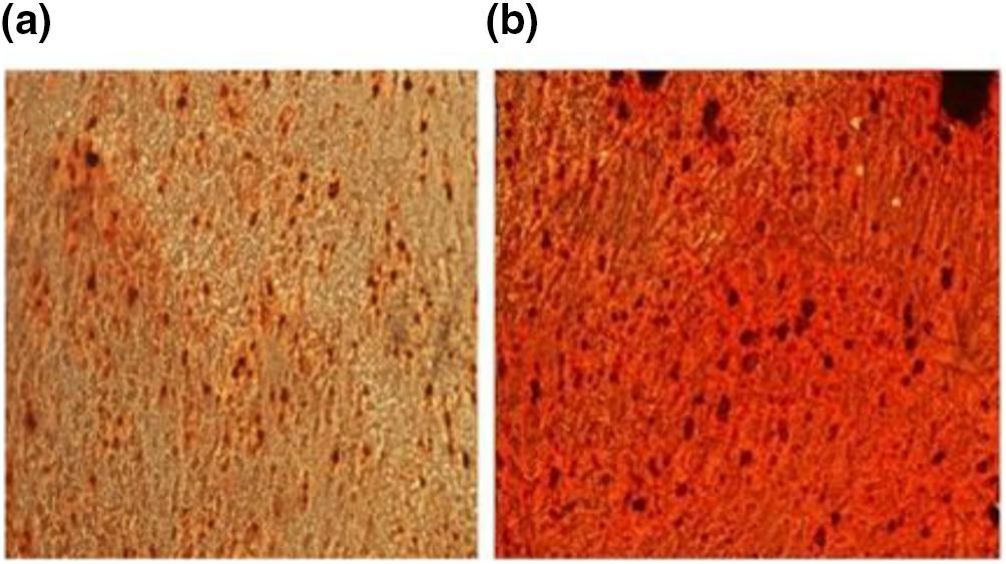
Fluorescence images of Saos2 cells on the surface of Mg‐0.4Ce/ZnO_2_ after 24 h. Brick red profile indicates calcium deposition by Saos2 cells: (a) control; (b) Mg‐0.4Ce/ZnO_2_

Figure 7a shows the calcium deposition as a marker of the late stage of osteogenesis, and this was measured by Alizarin red staining. Saos2 cells in contact with Mg‐0.4Ce/ZnO_2_ showed evidence of cell morphology similar to control cells after 1 day, as shown in Figure 7b. There was no evidence of cellular lysis and no inhibitory effects on cell growth were detected. Alizarin red stain was used to identify the calcium produced by Saos2 cells. The brick red deposits were proportional to the amount of calcium generated by the Saos2 cells. After 24 h the cells started to proliferate and showed good adherence on the surface of Mg‐0.4Ce/ZnO_2_. The cells were mostly elongated and polygonal in shape, which indicated that cells were well adhered, spread and proliferated. Polygonal‐shaped cells represent excellent adhesion and growth on the Mg‐0.4Ce/ZnO_2_ surface. The cells were found to be well adhered on the surface of magnesium nanocomposites and mineralisation (calcium deposition) by Saos2 cells was observed after 1 day in the form of a brick red colour. Both control and magnesium nanocomposites showed more calcium deposition by Saos2 cells [30, 38]. Therefore, the Mg‐0.4Ce/ZnO_2_ promotes cell proliferation without affecting the normal functioning of Saos2 cells. From the results of Alizarin red staining, it is found that Mg‐0.4Ce/ZnO_2_ exhibited biomineralisation in static culture conditions after 1 day. Although some previous studies have shown that Mg‐RE‐based materials do not have a significant cytotoxic effect, there remains a great deal of concern about the potential toxicity of RE elements.

## 4 CONCLUSIONS

In the present study, the microstructure, in vitro degradation, and biocompatibilities of Mg‐0.4Ce/ZnO_2_ were assessed by a haemolysis and cytotoxicity test. The rate of degradation of Mg‐0.4Ce/ZnO_2_ after the first, third, fifth, seventh, and 14th days showed controlled degradation after exposure in phosphate buffer solution. From the results of haemolysis, Mg‐0.4Ce/ZnO_2_ does not meet the conditions for biomaterials and so an additional constraint is needed to prevent RBC lysis. The results of MTT assay for the Mg‐0.4Ce/ZnO_2_ surface appears to have a cytotoxicity effect after 1 day due to an increase in pH. From the results of Alizarin red staining, Mg‐0.4Ce/ZnO_2_ exhibited extracellular matrix mineralisation in the static culture conditions and the nodules were stained intensively after 1 day of observation. As in vitro and other relevant studies are not sufficient for the validation of biocompatible materials, a further sequence of in vivo studies has been followed for the approval of any compounds/materials for therapeutic purposes.
